# Recent Advances in Sensing Strategies for the Detection and Discrimination of Tea Polyphenols

**DOI:** 10.3390/bios16100552

**Published:** 2026-10-02

**Authors:** Xinbo Sun, Taotao Li, Ying Wu, Zhanjun Liu, Zhiyuan Hu, Yuan Li, Songlin Yu, Xiangdong Qing, Shiquan Liu

**Affiliations:** Hunan Provincial Key Lab of Dark Tea and Jin-Hua, Hunan City University, Yiyang 413000, China

**Keywords:** tea polyphenols, sensors, optical sensing, electrochemical sensing, hyperspectral imaging

## Abstract

Tea polyphenols largely determine tea quality, flavor, and health benefits. Reliable detection of these compounds is therefore critical for quality control and process optimization. This review categorizes current sensing strategies into four types: optical, electrochemical, spectroscopic/nondestructive, and sensor arrays. These platforms differ in transduction mechanisms, sensitivity, and practical utility. Optical and electrochemical methods respond rapidly but are vulnerable to matrix effects. Spectroscopic techniques allow non-destructive analysis; their limitation lies in model generalizability across different instruments and sample sets. Sensor arrays, particularly when paired with machine learning, show strong potential for distinguishing structurally similar catechins and tracing tea origin and authenticity. Finally, the future development trends and potential application scenarios of tea polyphenols detection are discussed.

## 1. Introduction

Tea polyphenols (TPs) are a group of polyphenolic compounds naturally present in tea [1,2,3,4,5]. They consist primarily of catechins, flavonoids, anthocyanins, phenolic acids, and their derivatives. These compounds are major determinants of tea color, aroma, taste, and bioactivity. Catechins constitute the largest proportion of TPs and mainly include epigallocatechin (EGC), epicatechin (EC), epigallocatechin gallate (EGCG), and epicatechin gallate (ECG) [6,7,8,9]. Their catechol and pyrogallol moieties confer strong reducing power and radical-scavenging activity [10,11,12,13]. TPs possess multiple biological activities, including antioxidant, anti-inflammatory, anticancer, metabolic regulatory, and neuroprotective effects. These properties are primarily associated with the scavenging of reactive oxygen species, modulation of inflammatory signaling pathways, and regulation of apoptosis. TPs also contribute to the astringency and sweet aftertaste of tea infusion. During tea processing, they undergo oxidative polymerization to form pigments such as theaflavins and thearubigins, thereby directly affecting the liquor color and flavor characteristics of black and Oolong teas [14,15,16,17]. Differences in processing methods result in distinct retention levels and transformation pathways of TPs among tea categories [18,19,20]. Green tea is subjected to minimal enzymatic oxidation and therefore retains most of its original catechins, whereas the extensive oxidation occurring during black tea processing converts a large proportion of catechins into polymerized products [21,22,23,24]. Oolong tea exhibits an intermediate degree of oxidation. The growing use of TPs as natural antioxidants in foods, health products, and cosmetics has created a demand for accurate detection.

Analytical methods for TPs have been developed from conventional chemical assays to modern sensing technologies. Early analyses relied on spectrophotometric methods, particularly the Folin–Ciocalteu assay, which estimates total phenolic content indirectly via absorbance of a blue complex (phosphomolybdotungstate blue) formed by the reduction of Folin–Ciocalteu reagent by phenolic hydroxyl groups. For quantification of individual compounds, chromatographic approaches—particularly high-performance liquid chromatography (HPLC) coupled with UV or mass spectrometric detectors—have become the gold standard for separating and quantifying TPs. Advances in sensing principles and functional materials have led to the development of new analytical approaches [25,26,27,28,29,30]. Colorimetric and fluorescent probes constructed from carbon quantum dots and nanozymes can generate measurable color or luminescence changes upon interaction with TPs. Electrochemical methods employing modified electrode materials can facilitate electron transfer and improve the analytical sensitivity and stability of TP detection [31,32,33,34]. Meanwhile, nondestructive techniques, including hyperspectral imaging, near-infrared spectroscopy, and ultrasonic sensing, have been combined with chemometric models to enable rapid prediction of TP content and, in some cases, their spatial distribution within tea samples.

This review is organized from single-signal methods (optical and electrochemical) to intrinsic-signal methods (spectroscopic and nondestructive) and finally to multi-channel strategies (sensor arrays), reflecting the progression from simple quantification to multi-component discrimination.

## 2. Probe-Based Optical Sensing Strategies

Optical methods offer rapid identification and quantitative analysis of TPs by recording changes in color and fluorescence [35]. These methods rely on specific probes or chromogenic reactions that respond to TPs. Optical sensing systems based on colorimetric, fluorescent, and multimodal approaches have been developed, improving analytical sensitivity, selectivity, and practical utility.

### 2.1. Colorimetric Sensing

Colorimetric sensing of TPs mainly relies on two reaction mechanisms: the formation of colored complexes between polyphenols and metal ions, and the nanozyme-catalyzed oxidation of chromogenic substrates such as 3,3′,5,5′-tetramethylbenzidine (TMB).

Tea polyphenols contain abundant adjacent phenolic hydroxyl groups (catechol and pyrogallol moieties) that can chelate transition-metal ions, such as Fe^3+^ and Cu^2+^, through the formation of five-membered or six-membered coordination rings. This chelation alters the electronic distribution of the metal centers and gives rise to characteristic color changes, enabling visual detection of TPs. Jeong et al. developed a microbead-based colorimetric sensor consisting of yellow beads loaded with Fe^3+^ [36]. The detection requires no sample pretreatment and can be completed within 5 min using a smartphone for readout. Photographs were captured using a smartphone, and the extracted RGB values were used to quantify the polyphenol content. The limit of detection for tannic acid was 0.0415 mM, which was lower than that of the conventional Folin–Ciocalteu assay. Jiang et al. developed a homemade color-sensitive sensor for the quantitative estimation of tea polyphenols in green tea. Color features were selected using ant colony optimization, and an extreme learning machine model was subsequently established, yielding a validation correlation coefficient of 0.8035 and an RMSEP of 1.6003% [37].

Owing to their high enzyme-mimicking activity, high stability, and suitability for large-scale production, nanozymes have become common components in colorimetric sensors. A commonly used sensing system uses TMB as the chromogenic substrate and achieves the selective recognition of TPs by exploiting their regulatory or inhibitory effects on nanozyme-catalyzed reactions. A synthetic Fe_3_C/Fe-N-C catalyst was capable of catalyzing TMB oxidation in the absence of H_2_O_2_. In combination with pattern-recognition algorithms, this system enabled the detection and discrimination of four major catechins—EGCG, EGC, ECG, and EC [38].

Research on nanozyme-based polyphenol detection has investigated nanozymes with different catalytic properties, including Fe-N-C microflowers (Fe-N-C MFs), Au/Fe-N-C, Pt/Fe-N-C, Arac-Cu, bimetallic platinum–palladium nanoparticles (B-PtPdNPs), and BDC-Cu [39,40,41,42]. The immobilization of an Arac-Cu nanozyme within a hydrogel, together with a smartphone-based imaging device, enabled the rapid detection of TPs over a concentration range of 5–300 μM, with a limit of detection of 1.5 μM [39].

Yang et al. developed an enzyme/nanozyme hybrid strategy in which natural polyphenol oxidase (PPO) was combined with artificial nanozymes to construct a sensor array for identifying the six major categories of chinese tea [43]. They also developed a multimodal sensor array based on the BDC-Cu nanozyme. By introducing a photothermal signal for the first time, the system enabled the effective differentiation of white teas according to their storage years [44]. Colorimetric and fluorescent signals alone could not separate all TPs; the photothermal channel was needed to reach 100% discrimination. With advances in computational technologies and sensor arrays, TP analysis is gradually shifting from the determination of total polyphenol content toward multicomponent discrimination and tea-sample classification [38,44,45].

Fe^3+^-doped carbon dots (Fe-CDs) possess peroxidase-like activity, and TPs can act as their nanozyme cofactors. Based on this mechanism, a colorimetric sensor array was constructed and combined with PCA and HCA to achieve accurate discrimination of five classes of TPs and their mixtures, as well as authentication of different tea products [46]. Wu et al. constructed a visual and fluorescent dual-mode sensor array using nanoporphyrin and quantum dots. Through the selective combination of sensing units, the array enabled quantitative analysis of catechin enantiomers and accurately identified Xihu Longjing tea samples of different grades, storage times, and low-level adulteration from adjacent production regions [47].

With advances in computational technologies and sensor arrays, the introduction of novel recognition mechanisms further extends the discriminatory capability of colorimetric sensing. Hu et al. proposed a sensing strategy that integrates boronic acid-based sensors with pH indicators. Leveraging the reversible binding between boronic acid and the cis-diol structures of TPs and the resulting pH changes, this strategy enabled naked-eye colorimetric discrimination of six different TPs [48]. In addition, Duan et al. synthesized Rh-decorated Pd nanocubes via underpotential deposition-mediated synthesis, which exhibited enhanced peroxidase-like activity. The multichannel colorimetric sensor constructed on this basis enabled rapid discrimination of six TPs and nine real tea samples, demonstrating the potential of nanozyme morphology engineering in improving colorimetric sensing performance [49].

### 2.2. Fluorescent Sensing

Fluorescent sensing offers high sensitivity and temporal resolution, giving it potential for the detection of trace amounts of polyphenols. Carbon dots and carbon quantum dots (CDs) are among the most studied fluorescent probes in recent years. These nanomaterials can be prepared from natural biomass and combine environmental compatibility with useful optical and surface properties.

Thombre et al. used spent Oolong tea leaves as a precursor to prepare carbon quantum dots through a one-step microwave-assisted method [50]. The obtained CDs show strong and stable fluorescence which was quenched by TPs. Using these CDs as fluorescent probes, the EGCG concentrations in black tea and Oolong tea samples were determined to be 28.65 and 50.42 mg/L, respectively.

Wei et al. constructed a fluorescent sensing system based on competitive coordination and fluorescence recovery [51]. Initially, the fluorescence of CDs was quenched by Cu^2+^. After TPs were introduced, their phenolic hydroxyl groups preferentially coordinated with Cu^2+^, releasing the CDs and restoring their fluorescence. The method provided a linear response over the concentration range of 1–30 μg/mL, with a limit of detection of 0.31 μg/mL, and was suitable for determining total polyphenol content in different tea samples. The sensitivity is higher than that of colorimetric sensors, but the method requires buffer conditions and a spectrofluorometer, which limits its use for on-site analysis.

Rare-earth-metal complexes constitute another important class of fluorescent sensing materials. Gorai et al. developed a terbium-ion-based paper photoluminescent sensor for detecting gallate-derived polyphenols in green tea [52]. Tb^3+^ and a ligand capable of specifically recognizing gallate groups were incorporated into filter paper immobilized with a supramolecular gel, thereby forming a ternary Tb^3+^–ligand–polyphenol complex. The presence of target polyphenols induced a synergistic luminescence response. This sensing platform is inexpensive and requires only a small sample volume. The detection limit for gallate-derived polyphenols was 300–360 nM, and the cost per assay was estimated at €0.0015. The sensor responds only to gallate derivatives and cannot detect non-gallate catechins. A water-soluble phenylboronic-acid-functionalized perylene diimide probe can form dynamic boronate esters with the cis-diol groups of TPs. Because structural differences among catechins induce distinct degrees of probe self-assembly and fluorescence quenching, EC, ECG, EGC, and EGCG can be discriminated when the fluorescence responses are combined with linear discriminant analysis [53]. This system has only been tested with catechin standards in buffer solutions; whether it works well in real tea samples needs further study.

Tb^3+^ and Eu^3+^ co-doped Zr(IV) metal–organic frameworks have also been employed to construct fluorescent sensors for TP detection [54]. Taking advantage of the fluorescence derived from pyrene conjugated groups, a fluorescent test paper was prepared and exhibited a notable fluorescence enhancement response toward TP solutions, with a detection limit of 5 × 10^−6^ mg/mL [55]. Fluorescent sensing can also be coupled with other analytical techniques. Dopamine reacts rapidly with TPs containing resorcinol structures to generate fluorescent azamonardine products. When combined with chromatographic separation, this strategy enables the identification of structurally similar TPs [26]. Poly(p-phenyleneethynylene) (PPE) exhibits fluorescence that is modulated by metal-ion coordination and interchain π–π stacking. TPs could be detected via the fluorescence change of PPE induced by their binding [56].

HPLC remains the standard method for quantifying individual catechins, with recovery rates of 95–105% and RSD below 2%. Optical sensors are simpler and faster but less accurate for individual polyphenol quantification. They often respond to total polyphenols or a subclass rather than individual compounds. The Fe^3+^-loaded microbead sensor detected tannic acid with a detection limit of 0.0415 mM [36], and the carbon dot/Cu^2+^ probe reached 0.31 μg/mL for total polyphenols [51]. These values are adequate for rapid screening but do not match the sensitivity of HPLC-MS for trace analysis. Colored tea infusions also interfere with optical signals more than they do with HPLC, where chromatographic separation removes interfering substances.

Colorimetric sensors are easy to use and allow visual readout without instrumentation, but they are less sensitive than fluorescent probes, and colored tea infusions interfere with the signal. Fluorescent sensors respond faster and reach lower detection limits, but they suffer from photobleaching, are sensitive to pH and temperature, and background fluorescence from the sample can interfere. Most fluorescent probes respond to total polyphenols or a subclass rather than individual catechins. Representative optical sensing strategies and their principal characteristics are summarized in Table 1.

## 3. Electrochemical Sensing Strategies

Electrochemical sensing technologies have attracted attention for the rapid detection of TPs because of their operational simplicity, rapid response, and ease of integration into portable devices.

### 3.1. Direct Electrochemical Oxidation

The electrochemical detection of TPs relies on the oxidation of phenolic hydroxyl groups at the electrode surface. When a suitable anodic potential is applied, TPs undergo irreversible electrochemical oxidation, transferring electrons to the electrode and generating a Faradaic current. The current intensity is proportional to the TP concentration. To enhance electron transfer kinetics and mitigate electrode fouling caused by oxidative polymerization of phenolic products, various electrode modification strategies have been developed. This reagent-free format allows simple, label-free detection.

Cyclic voltammetry (CV) and differential pulse voltammetry (DPV) are common techniques for direct electrochemical detection. They effectively discriminate among different tea samples [57]. Bare electrodes, such as glassy carbon electrodes (GCEs), are hindered by slow electron transfer and surface passivation from oxidation products. These drawbacks lead to poor signal reproducibility and limited sensitivity. To overcome these limitations, chemically modified electrodes are used for tea polyphenol detection. In situ growth of AuNPs on eggshell membrane (ESM) improved electrode conductivity and surface area. This enhancement led to higher oxidation peak currents for gallic acid (GA), caffeic acid (CA), and catechin hydrate (CH) [58]. Shi et al. prepared an electrochemical sensor for TPs by loading iron nanoparticles onto cassava fibers. The sensor exhibited a linear response over the concentration range of 3.5–31.5 µM, with a limit of detection of 0.1 µM [59]. Cheng et al. fabricated microelectrodes of different sizes using electrohydrodynamic printing technology. The 70-µm microelectrode showed the highest sensitivity toward low concentrations of TPs, with a limit of detection of 0.22 μmol/L and recoveries of 95.2–105.0% in green tea samples [60]. The recoveries are comparable to those of HPLC.

### 3.2. Enzyme-Based and Enzyme-Mimetic Electrochemical Sensing

Two categories of biocatalytic materials have been employed to amplify electrochemical signals: (i) natural oxidases such as tyrosinase and polyphenol oxidase, which catalyze the oxidation of phenolic substrates with high substrate specificity; and (ii) nanozymes—nanomaterials with enzyme-like catalytic activities, including peroxidase-like and oxidase-like activities—that offer higher stability and lower cost compared to natural enzymes. Beyond direct oxidation, enzyme-like catalytic materials have been employed to amplify electrochemical signals with enhanced selectivity. Materials with enzyme-like catalytic activity have been employed to amplify electrochemical signals from TP oxidation. Commonly used catalytic components include nanomaterials with peroxidase-like (POD-like) or oxidase-like (OXD-like) activity, as well as immobilized natural oxidases.

Nanozymes are nanomaterials with enzyme-like catalytic activity. Like natural enzymes, they lower the activation energy of a reaction and provide catalytic sites for substrate conversion. Unlike natural enzymes, they are more stable under harsh conditions, easier to produce at scale, and less expensive. These features make nanozymes attractive for electrochemical detection of TPs. In electrochemical sensing, nanozymes are usually immobilized on the electrode surface, where they catalyze the oxidation of phenolic substrates and generate an amplified current signal. Datta et al. developed an eggshell-membrane-based biosensor by preparing AuNPs through an in situ reduction method. The AuNPs and tyrosinase (Tyr) were co-immobilized on the membrane, which was subsequently assembled onto the surface of a glassy carbon electrode. The limits of detection for gallic acid, caffeic acid, and catechin hydrate were 1.707, 0.752, and 0.714 μM, respectively. The sensor could also be reused without loss of sensitivity [58]. The detection limits are higher than those of direct oxidation on modified electrodes, but the enzyme provides better selectivity toward individual phenolic substrates. The immobilized enzyme is sensitive to pH and temperature, which limits its long-term stability.

Perone et al. immobilized polyphenol oxidase (PPO) extracted from plant waste onto cellophane membranes and integrated the membranes with a Clark electrode for the determination of polyphenols in tea samples. The analytical results were consistent with those obtained using the conventional Folin–Denis method [61]. In addition, an electrochemical sensor for TPs was constructed by co-depositing graphene nanoribbons (GNRs), silver nanoparticles (AgNPs), and polyphenol oxidase (PPO) onto the surface of a graphite electrode [62]. Di Giulio et al. developed an electrochemical sensor for gallic acid (GA) using ultra-small pyramidal platinum nanoparticles (PtNPs, ≈4 nm) deposited onto a glassy carbon electrode by a simple drop-casting method [29]. The sensor required only 2.7 μg of PtNPs per electrode and employed multiple pulse amperometric detection to alleviate electrode fouling caused by GA oxidation products. The detection limit was 16 μM, higher than that of the cassava fiber–iron electrode, but the sensor is more stable than enzyme-based electrodes, and the results were validated by HPLC.

Alongside noble metals and carbon nanomaterials, layered double hydroxides (LDHs) have emerged as promising supports for enzyme-based electrochemical sensors, owing to their favorable biocompatibility and high specific surface area. Soussou et al. immobilized tyrosinase onto a CoAlSO_4_ LDH film coated on a screen-printed gold electrode via cross-linking, and constructed an amperometric polyphenol biosensor that exhibited a linear response over the concentration range of 0–2.4 µM [63]. The introduction of redox mediators offers an alternative route to improve the selectivity and sensitivity of electrochemical detection. Sen et al. reported that a chloramine-T modified electrode enabled the detection of TPs with a limit of detection of 0.674 mg/L [64].

Screen-printed electrodes (SPEs) are fabricated by depositing conductive inks onto insulating substrates through a screen-printing process, thereby integrating the working, counter, and reference electrodes into a three-electrode system. Their advantages include low cost, suitability for mass production, portability, and disposability, rendering them suitable for rapid on-site analysis.

Marlin et al. modified a screen-printed carbon electrode (SPCE) by drop-casting an iron tetrasulfonated phthalocyanine (FeTsPc) solution onto its surface, thereby constructing an SPCE/FeTsPc sensor [65]. The oxidation potential of catechol at the modified electrode was lower than that observed at the unmodified SPCE, demonstrating the good electrocatalytic activity of FeTsPc. The limit of detection and limit of quantification were 0.6 and 2.0 µmol/L, respectively. They also reported the use of an FeTsPc-modified SPCE for the determination of polyphenols in yerba mate, confirming the reliability and reproducibility of this approach [65]. The electrode is cheap and disposable, making it suitable for portable devices, but its reproducibility is usually lower than that of conventional electrodes.

### 3.3. Flow Injection Analysis Coupled with Electrochemical Detection

Flow injection analysis (FIA) automates sample introduction by injecting discrete sample plugs into a flowing carrier stream for online mixing, reaction, and detection. Coupling FIA with electrochemical detectors can overcome some of the limitations of conventional batch methods, including high reagent consumption and long analysis times, and is therefore suitable for the rapid screening of large numbers of samples. In one study, a glassy carbon electrode modified with graphitized mesoporous carbon (GCE/GMC), combined with differential pulse voltammetry, was used for the simultaneous determination of three dihydroxybenzene (DHB) isomers—1,4-, 1,2-, and 1,3-DHB—with limits of detection of 0.91, 1.31, and 0.67 mM, respectively [57].

Liang et al. proposed a flow batch analysis system (FBAS) based on a diazotization–coupling (DC) reaction for the determination of TPs [66]. By integrating the automation advantages of FIA with the selectivity of chemical derivatization, the system reduced interference from nonphenolic substances. It achieved a limit of detection of 0.215 μg/mL and relative standard deviations (RSDs) of 0.326–0.659%.

Electrochemical sensors are faster, cheaper, and more portable than HPLC, but their accuracy and reproducibility are generally lower. The tyrosinase/AuNP/eggshell membrane electrode detected gallic acid, caffeic acid, and catechin hydrate with detection limits of 1.707, 0.752, and 0.714 μM, and the results agreed well with HPLC [58]. The PtNP-modified electrode detected gallic acid in green and black tea with results comparable to HPLC [29]. These sensors cannot separate individual catechins in a mixture without prior chromatographic separation. HPLC remains the standard method for quantifying individual catechins. Electrochemical sensors are better suited for rapid on-site screening of total polyphenol content, while HPLC is preferred when individual compound quantification is required.

Direct oxidation is simple and requires no reagents, but bare electrodes foul easily and give poor reproducibility, and they cannot distinguish individual polyphenols. Enzyme-based electrodes are more selective because the enzyme only reacts with specific substrates, but the immobilized enzyme is unstable and sensitive to pH and temperature. Nanozyme-modified electrodes are more stable than enzyme-based ones and still provide catalytic amplification, but batch-to-batch variation of the nanomaterial remains a problem. Screen-printed electrodes are cheap and disposable, making them suitable for portable devices, but their reproducibility is usually lower than that of conventional electrodes. For rapid total polyphenol screening, direct oxidation is sufficient. When selectivity is needed, enzyme-based electrodes are preferred. For portable on-site use, nanozyme-modified or screen-printed electrodes are more practical. Representative methods and their main characteristics are summarized in Table 2.

## 4. Spectroscopic and Nondestructive Sensing

Unlike optical sensing methods that rely on specific probes or chromogenic reactions, spectroscopic and nondestructive detection techniques primarily acquire the intrinsic reflection, absorption, and physical propagation signals of tea leaves or tea infusions. Chemometric or machine-learning models are subsequently used to establish relationships between these signals and TP content. Such methods generally require neither complex chemical reactions nor destructive sample preparation and are therefore suitable for fresh-leaf evaluation, finished-tea grading, and process monitoring.

### 4.1. Visible Spectroscopy and Near-Infrared Spectroscopy

Visible spectroscopy measures the absorption or reflectance of light in the visible region (380–780 nm), which primarily reflects electronic transitions of chromophores such as tea pigments and polyphenol–metal complexes. Near-infrared spectroscopy (NIR, 780–2526 nm), in contrast, detects overtones and combination bands of fundamental molecular vibrations, mainly associated with O–H, C–H, and N–H bonds in tea constituents. Both techniques reflect changes in chemical groups and tissue structures in tea, making them common signal sources for the nondestructive prediction of TPs. Research in this area has gradually shifted from low-cost spectral acquisition toward spectral-range fusion, feature selection, and dynamic process monitoring.

A fresh-tea-leaf constituent detection device based on multichannel spectral data in the range of 700–1000 nm and a random forest model was capable of providing multiple quality indicators in real time. However, the coefficient of determination for the TP prediction model was only 0.28 [69]. The device is portable and requires no chemical reagents, but the prediction accuracy for TP content is lower than that of NIR models built on larger datasets. By fusing visible/short-wave near-infrared and long-wave near-infrared spectra, information associated with chromophores, auxochromes, and hydrogen-containing functional groups could be simultaneously utilized. The resulting least-squares support vector regression (LS-SVR) model achieved a prediction correlation coefficient of 0.893 for TP content in fresh tea leaves, superior to models based on either spectral range alone [70]. An infrared fiber sensor developed for TP detection achieved a sensitivity of 1.52 a.u./(vol%) [71]. Near-infrared spectroscopy has also been extended to the monitoring of tea-processing operations. During Pu-erh tea fermentation, standard normal variate (SNV) transformation was applied to reduce spectral noise, after which competitive adaptive reweighted sampling (CARS) was used to select 52 informative variables from the original spectra. A partial least-squares (PLS) model based on these variables enabled the nondestructive prediction of TP content in fermented tea liquor [72]. The model achieved Rp = 0.9088 and RPD = 2.372, higher than that of the multichannel spectral device. The method requires no sample pretreatment and is suitable for process monitoring.

The recent development of near-infrared spectroscopy for TP detection has centered on the optimization of intelligent algorithms. Xu et al. combined three wavelength selection methods—competitive adaptive reweighted sampling (CARS), uninformative variable elimination (UVE), and successive projections algorithm (SPA)—with partial least squares regression and principal component regression to establish quantitative models for the ratio of polyphenols to free amino acids (TP/FAA) in Wuyi Rock Tea [73]. Yin et al. integrated four wavelength selection algorithms, namely CARS, BOSS, VCPA-GA, and VCPA-IRIV, and developed synchronous prediction models for TPs, amino acids, caffeine, and major catechin monomers (EGCG and ECG) based on 233 fresh leaf samples of different varieties and tenderness grades [74]. Qiu et al. acquired near-infrared spectra and determined the contents of theanine, TPs, water extracts, and soluble sugars, and established a quality component prediction model based on multiple spectral features, enabling rapid detection of quality components in green tea [75]. Ye et al. demonstrated that Fourier transform near-infrared (FT-NIR) spectroscopy combined with machine learning algorithms enables synchronous prediction of TP and EGCG contents in tea leaves [76].

### 4.2. Hyperspectral Imaging-Based Sensing

**Figure 1:**
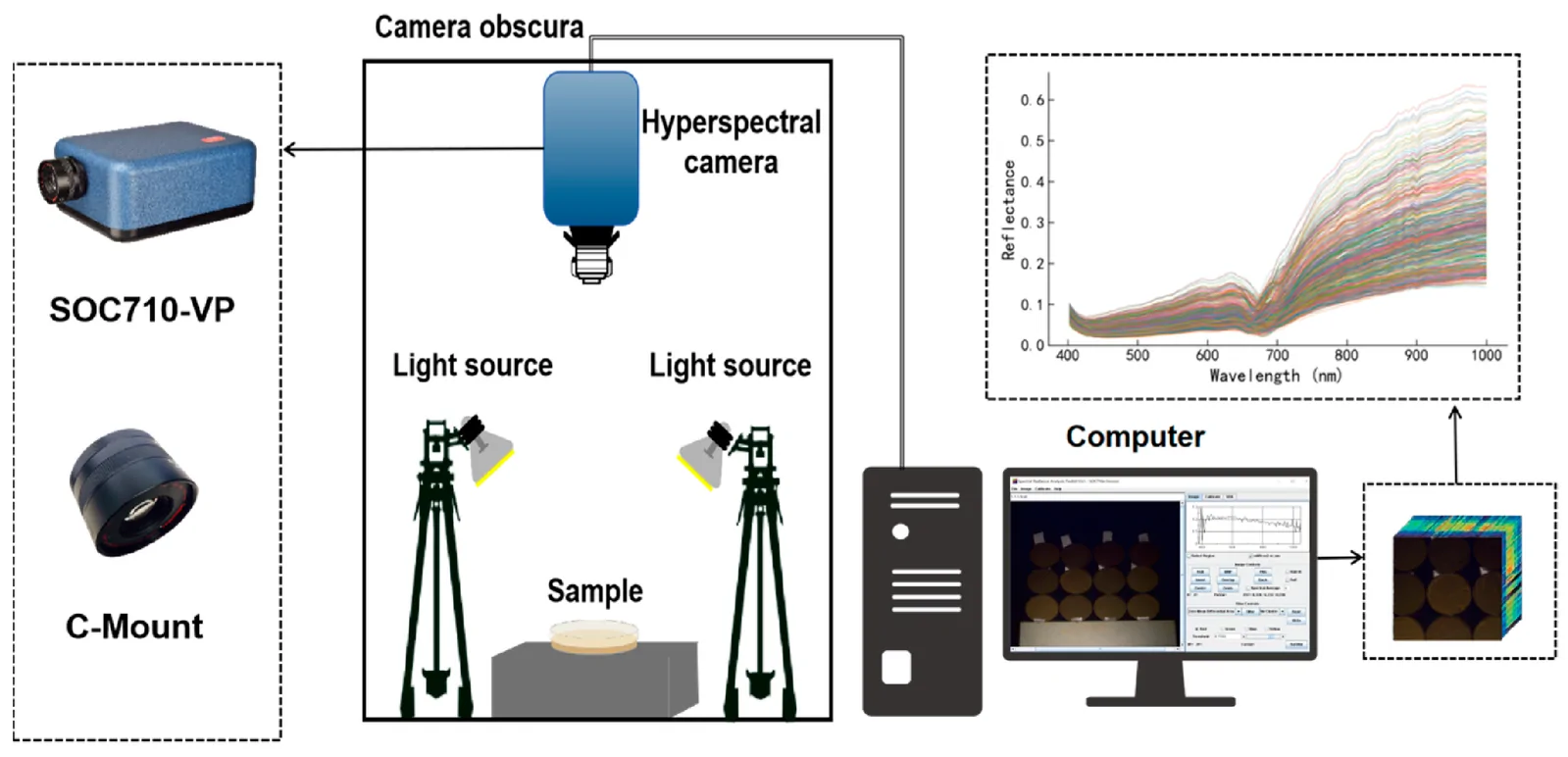
The hyperspectral imaging system. Reprinted with permission from ref. [79]. Copyright 2024 MDPI.

Hyperspectral imaging (HSI) integrates spatial distribution and image-texture information with continuous spectral data, allowing the internal chemical composition and external morphological characteristics of tea samples to be characterized simultaneously. The hyperspectral imaging system is shown in Figure 1.

Ensemble modeling can reduce the dependence of an individual regression algorithm on specific sample characteristics. For the prediction of TPs in Tibetan tea, a stacking strategy was used to integrate multiple tree-based models. The resulting model achieved a coefficient of determination of 0.9625 for the prediction set, outperforming the individual regression models [77]. The accuracy is higher than that of NIR-based models, but hyperspectral imaging requires more complex instrumentation and data processing.

To address the limited texture differences observed at low image resolutions and the excessive feature dimensionality encountered at high resolutions, multiscale wavelet decomposition, gray-level co-occurrence matrix (GLCM) texture features, and wavelet texture features were integrated. A support vector regression (SVR) model based on the fused features was then established for predicting TP content in yellow tea. The model achieved an R^2^ of 0.871 and an RMSE of 0.830 for the calibration set, together with an R^2^ of 0.838 and an RMSE of 0.896 for the validation set. Its predictive accuracy was superior to that of models based on individual feature types and to that of the partial least-squares regression (PLSR) model [78].

For different categories of tea samples, the compatibility between feature-processing methods and model structures directly affects predictive performance. An improved Pied Kingfisher Optimization algorithm was used to optimize the parameters of an SVR model for detecting TP content in Fu brick tea. The resulting model achieved an R^2^ of 0.9152 and an RPD of 3.4345 for the test set, outperforming K-nearest-neighbor regression, back-propagation neural networks, and other optimized SVR models [79]. For Tieguanyin tea, the combination of first-derivative preprocessing, CARS, and a random forest algorithm produced an R^2^ of 0.938 and an RPD of 4.474 for the prediction set [80].

Feature wavelength extraction combined with model optimization has been shown to enhance both the accuracy and sensitivity of hyperspectral imaging-based sensing. Long et al. proposed a refined feature wavelength method named IBS-CARS-Fusing to extract effective wavelengths from visible-near-infrared (VNIR) and short-wave near-infrared (SWIR) hyperspectral images [81]. With this method and a kernel ridge regression (KRR) model, the predictive determination coefficient (R^2^_p_) for TPs was improved by 15.5% [81]. Li et al. identified the two-band normalized difference spectral index based on 1673/1660 nm as a key spectral feature for TP detection, achieving a predictive R^2^_p_ of 0.785 [82].

### 4.3. Ultrasonic Sensing

Ultrasonic sensing is based on the principle that changes in solution composition affect the propagation velocity of acoustic waves. By measuring the ultrasonic time of flight and incorporating temperature information, a quantitative relationship can be established between sound velocity and TP concentration.

A detection system based on capacitive micromachined ultrasonic transducers (CMUTs) integrated ultrasonic transmission, reception, and temperature monitoring into a single platform. Sound velocity was calculated and used to predict TP concentrations, with a maximum relative error of 3.7% [83]. This study marks the first use of CMUT devices in TP quantification. Ultrasonic methods are insensitive to infusion color and scattering and require no chromogenic agents, but their limitation lies in the non-specificity of sound velocity—temperature, sugars, and other co-solutes all affect acoustic behavior. Reliable detection therefore depends on rigorous temperature correction and calibration against real sample matrices.

Visible spectroscopy mainly reflects electronic transitions of chromophores and gives limited information for polyphenol quantification. Near-infrared spectroscopy detects overtones and combination vibrations of O-H, C-H, and N-H bonds. It works well for rapid, non-destructive prediction of total polyphenol content, but the calibration model often does not transfer well to other instruments or sample sets. Hyperspectral imaging adds spatial information to spectral data. It can predict polyphenol content and show its distribution across the sample, but the cost is high data dimensionality, redundant information, and the need for careful feature selection and modeling. Ultrasonic sensing is not affected by sample color or turbidity and needs no reagents, but its signal is non-specific because temperature, sugars, and other dissolved substances also change the sound velocity. For routine quality control, near-infrared spectroscopy is the most practical. For detailed grading and spatial analysis, hyperspectral imaging is the better choice. For online monitoring of liquid samples, ultrasonic sensing is suitable. Representative methods and their principal characteristics are summarized in Table 3.

## 5. Sensor Arrays and Multisource Information Fusion Strategies

A single sensing signal can generally reflect changes in TP content but is often insufficient to distinguish structurally similar monomers, such as EGCG, EGC, ECG, and EC. It also has difficulty resolving overlapping responses caused by the coexistence of multiple constituents in tea samples. Rather than requiring each recognition element to exhibit absolute selectivity toward a particular TP, sensing strategies exploit cross-reactive responses generated by different materials, reaction channels, wavelengths, reaction times, or signal modalities. Chemometric and machine-learning methods are then used to extract characteristic fingerprints of TPs. This strategy has expanded TP detection from single-content determination to monomer discrimination, mixture analysis, and the identification of tea varieties, storage years, geographical origins, and adulteration.

### 5.1. Nanozyme and Enzyme–Nanozyme Sensor Arrays

Nanozyme-based arrays use several nanozymes with different catalytic properties as sensing units. Each nanozyme catalyzes a chromogenic reaction at a different rate, and TPs inhibit this reaction to different extents depending on their structure. When multiple nanozymes are combined, each TP produces a unique response pattern across the array. A paper-based array simultaneously utilized the polyphenol oxidase-like and peroxidase-like activities of nanozymes to generate distinct color responses to seven TPs. Combined with linear discriminant analysis (LDA) and hierarchical cluster analysis (HCA), the system enabled TP recognition, differentiation of Longjing tea according to production region, and adulteration identification [85]. Another array based on nanozymes with laccase-like and peroxidase-like activities employed a concentration-independent classification model to reduce interference caused by variations in sample concentration. The system was applied to discriminate White Peony teas with different storage years and identify adulterated samples [45].

Increasing the number of reaction channels can enhance the resolving power of sensor arrays. Fe_3_C/Fe-N-C nanozymes catalyzed the oxidation of TMB, and cascade chromogenic channels amplified the response differences among EGCG, EGC, ECG, and EC, enabling the discrimination of these four catechins [38]. When Pt and Au were separately deposited onto Fe-N-C materials, the two noble metals differentially modulated nanozyme catalysis and the inhibitory effects of TPs, generating complementary responses. The resulting three-channel array could distinguish TP monomers and identify commercial green tea beverages [40].

Instead of using multiple sensing materials, a single material can also generate multichannel signals. A bimetallic PtPd nanozyme catalyzed TMB oxidation without requiring an additional oxidant and produced multiple absorption peaks at 370, 450, and 650 nm. Different TPs exerted distinct effects on the signals at these wavelengths, allowing a single probe to generate multidimensional visual fingerprints. In combination with smartphone-based RGB analysis and LDA, the system enabled the classification of TPs, polyphenol mixtures, and green tea samples differing in type, freshness, and degree of adulteration [41]. In another strategy, five characteristic wavelengths were combined with three reaction times to create 15 sensing units. The incorporation of time-resolved responses increased the amount of information obtainable from a single nanozyme-catalyzed reaction. Combined with a support vector machine (SVM), this array recognized TPs at different concentrations and mixing ratios and classified green tea categories and adulterated samples, with a limit of detection of 5 μM [42].

Combining natural enzymes with nanozymes expands the diversity of array responses. Natural polyphenol oxidase and nanozymes can catalyze the reaction between TPs and 4-aminoantipyrine, generating cross-reactive color responses because of their different catalytic characteristics. When combined with a machine-learning-based dual-output model, the system simultaneously predicted the class and concentration of TPs in unknown samples. It was also used for the hierarchical identification of the six major categories of chinese tea and more specific tea varieties [43]. The enzyme component provides better selectivity. It is sensitive to pH and temperature, which limits its long-term stability.

Enhancing both the recognition dimensionality and anti-interference capability of nanozyme-based sensor arrays represents a key route to broadening their practical applicability. Li et al. developed a trimetallic nanozyme with switchable enzyme-mimetic reactions that exhibits substrate-dependent differential catalytic activities toward various phenolic substrates, thereby enabling interference-free discrimination of TPs [86]. Chen et al. employed MnOOH as the sole sensing element, which reduced the complexity of array fabrication. Taking advantage of the differences in reducing capacity among TPs, this system generated distinctive kinetic absorbance curves and achieved efficient discrimination of individual polyphenols at varying concentrations, distinct polyphenolic species, and their multi-component mixtures [87]. Phenylboronic acid functionality can boost both catechol-binding affinity and the catalytic efficiency of nanozymes. Bi et al. exploited this principle in a DBA-Cu nanozyme-based sensor array. The boronic acid groups on this array enabled highly efficient TPs detection [88]. Machine learning offers a powerful tool for signal processing. When applied to sensor arrays, it significantly improves recognition performance [89]. Ren et al. combined a nanozyme sensor array with ML algorithms, achieving precise identification of five TPs [90].

**Figure 2:**
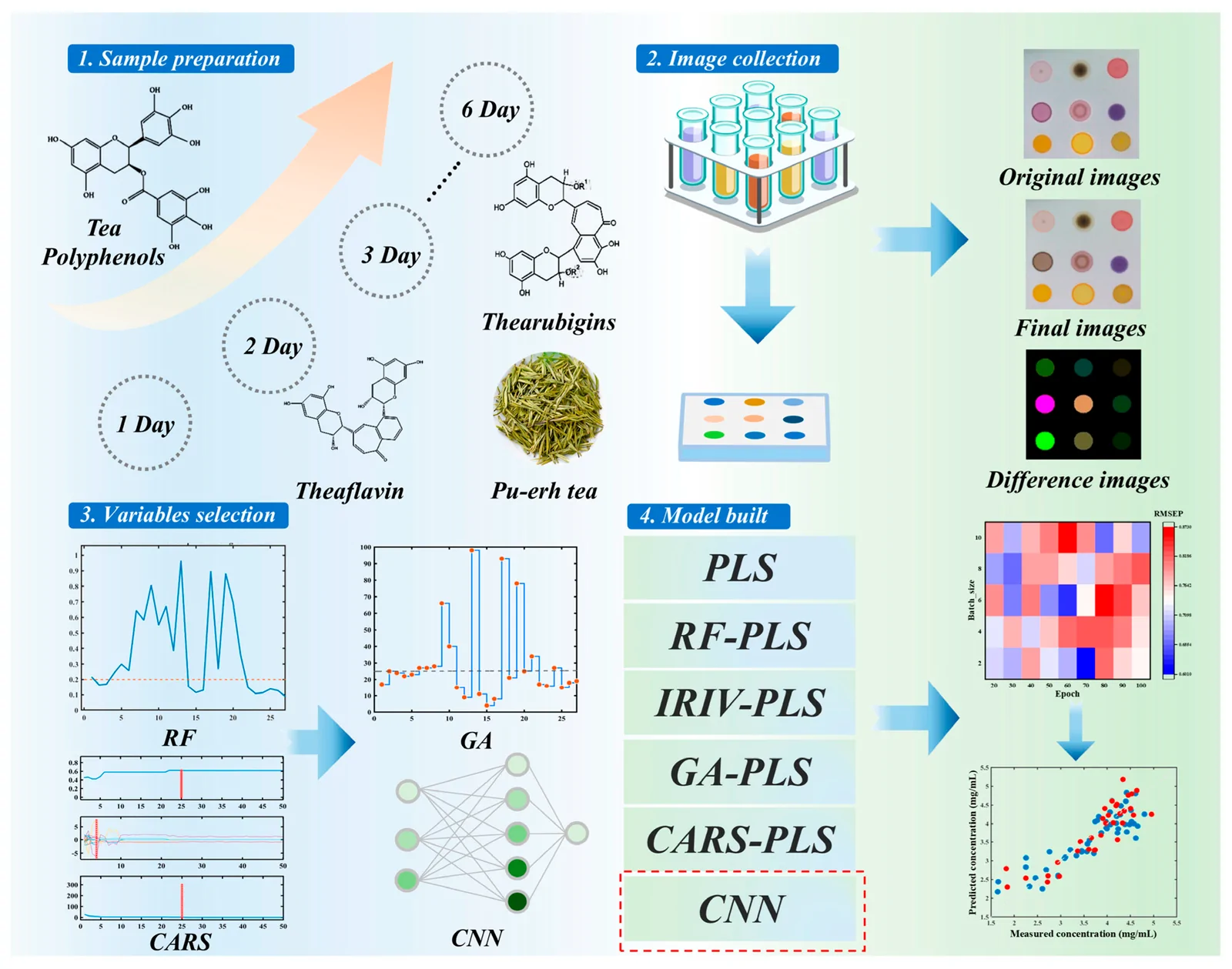
Schematic overview of polyphenol detection using CSA and chemometrics. Reprinted with permission from ref. [91]. Copyright 2024 MDPI.

A nanozyme-based array simultaneously generated colorimetric, fluorescent, and photothermal signals. The colorimetric and fluorescent channels alone were insufficient for accurate discrimination, whereas the introduction of the photothermal signal enhanced the ability to identify TPs. Combined with a random forest model, the array could also distinguish white teas according to storage year [44]. This system advanced sensor-array design from increasing the number of similar color channels to integrating signals based on different physical mechanisms. Its improved performance arose from signal complementarity rather than the absolute selectivity of any individual channel. Liu et al. developed a printed colorimetric sensor array (CSA) composed of eight porphyrin dyes and one pH dye for predicting total polyphenol content in Pu-erh tea liquid during microbial fermentation [91]. Under optimized fermentation conditions (determined by Box–Behnken sampling and response surface methodology), the polyphenol degradation rate reached 66.146%. Among six chemometric approaches compared, convolutional neural network (CNN) achieved the best predictive performance for total polyphenol content, demonstrating that the CSA-CNN system can effectively predict the fermentation degree of microbial-fermented Pu-erh tea by measuring polyphenol degradation (Figure 2).

### 5.2. Molecular Recognition and Single-Molecule Sensing

Cross-reactive sensor arrays rely on multiple nonspecific signals to construct characteristic fingerprints, whereas molecular recognition strategies attempt to establish a direct relationship between TP structures and sensing responses. A water-soluble phenylboronic-acid-functionalized perylene diimide probe exploited differences in boronate ester formation and probe aggregation induced by different catechins. These interactions generated structure-dependent fluorescence-quenching responses, which were combined with LDA to distinguish EC, ECG, EGC, and EGCG [53]. By integrating molecular recognition with self-assembly-based signal amplification, this system converted subtle structural differences into classifiable ensemble fluorescence signals. However, its responses may be influenced by analyte concentration, aggregation state, and solution conditions.

Nanopore sensing has advanced TP recognition to the single-molecule level. A phenylboronic-acid-functionalized nanopore recorded ionic-current events generated when individual TP molecules translocated through or interacted with the nanopore. Differences in hydroxylation patterns, galloylation, and stereochemistry produced reproducible ionic-current fingerprints. With machine-learning-assisted classification, the platform achieved an identification accuracy of 96.9% for eight structurally similar catechins. It also enabled TP profiling in tea samples within 30 min and the monitoring of interactions between TPs and metal ions [92]. The nanopore approach provides single-molecule resolution and high accuracy. It requires specialized nanopore fabrication and is not easily adapted for routine on-site analysis.

### 5.3. Chemometrics and Multisource Sensor Information Fusion

Intelligent sensing depends not only on novel sensing materials but also on the extraction of tea-polyphenol-related information from complex signals. Research in this field has progressed from feature selection within a single sensor to cross-sensor fusion involving spectral, image, aroma, and process-parameter information.

Effective dimensionality reduction of individual sensing signals can minimize irrelevant and redundant information. Features obtained using a color-sensitive sensor were selected using an ant colony optimization algorithm and subsequently combined with an extreme learning machine to establish a predictive relationship between green tea sensor responses and TP content [37]. This study extracted informative variables from indirect color responses, demonstrating that feature optimization can enable nonspecific sensing signals to be used for TP quantification. The prediction correlation coefficient was 0.8035, indicating that although indirect signals have potential for rapid detection, their model performance may be more susceptible to differences among samples than methods based on direct chemical or spectroscopic responses.

Hyperspectral data fusion simultaneously exploits spectral and spatial information. The integration of multiscale wavelet coefficients, gray-level co-occurrence matrix texture features, and wavelet texture features provided a more comprehensive characterization of yellow tea samples than any individual feature type. The SVR model established using the fused features outperformed both single-feature models and the PLSR model [78].

Cross-sensor fusion overcomes the limitations of relying on a single signal source. An electronic nose provides time- and frequency-domain features associated with volatile constituents, whereas hyperspectral imaging provides spectral and spatial information. Fusion of the two data sources enabled the establishment of a cross-category model for evaluating TPs in black, green, and yellow teas. Following feature-importance selection and XGBoost modeling, the fusion model achieved an R^2^ of 0.998, outperforming models constructed from the features of either individual sensor [93,94,95].

Multisource fusion has also been applied to process monitoring. A nano-optical sensor combined with near-infrared spectroscopy simultaneously characterized changes in volatile components and TP consumption during fermentation and was used for the in situ evaluation of the fermentation degree of tea extracts subjected to ultrasound-assisted treatment [96]. Rather than selectively detecting a specific TP, this approach linked a quantitative TP model with aroma sensing and processing parameters, allowing the sensing results to reflect the fermentation state.

Because a single sensing signal is generally insufficient to distinguish structurally similar TPs and resolve multicomponent responses in complex tea samples, sensing strategies based on cross-reactivity, multisignal fusion, and molecular recognition have gradually emerged. Nanozyme-based arrays rely on cross-reactive catalytic responses and can discriminate structurally similar catechins. One signal channel is often not enough; extra channels such as photothermal or fluorescent signals are needed. Enzyme/nanozyme hybrid arrays combine the selectivity of natural enzymes with the stability of nanozymes. The enzyme part is still sensitive to pH and temperature. Molecular recognition and nanopore-based approaches give direct structure–response relationships and can identify individual catechins, but they need specialized probes or nanopore fabrication. Multisource fusion combines spectral, image, or process data to improve prediction accuracy. Different data sources vary in scale, noise level, and information content, so integration requires careful preprocessing and feature selection. Across all array strategies, large datasets are needed for model training, and model generalizability across different sample sets and instruments remains to be validated.

## 6. Conclusions and Future Perspectives

TPs are key bioactive components in tea, which determine the unique quality and flavor of tea. Accurate and portable analytical technology for TPs detection are therefore of considerable significance to the tea industry. This review covers four types of sensing strategies for TPs. The detection of TPs is transitioning from simple content quantification to rapid, portable, non-destructive, and intelligent analysis.

Optical probes are straightforward and fast, though their sensitivity can vary widely depending on the probe design. Electrochemical methods offer speed and reagent-free operation, but electrode fouling and poor reproducibility remain unresolved. Spectroscopic tools allow non-destructive measurement; their accuracy, however, is tied to model quality and transferability across instruments. Sensor arrays collect multi-channel data and, with the help of machine learning, enable rapid screening of large sample batches. The main difficulty lies in extracting reliable information from complex, overlapping signals. When machine learning and multisource fusion are combined, the efficiency of TP signal utilization improves substantially. This combination not only permits accurate identification of individual catechins but also extends the analytical scope—from simple content measurement to the discrimination of tea varieties, production years, geographical origins, and adulteration.

Future developments should go beyond mere compound identification and move toward application-driven scenarios: online and non-destructive quality assessment of tea leaves, discrimination of production years and geographical origins, and precise recognition of individual TPs. For quality evaluation and origin or vintage authentication of bulk tea samples, non-destructive spectroscopy—visible/near-infrared or hyperspectral imaging—can serve as a rapid first-line tool to acquire polyphenol content and related quality indicators, followed by chemometric or machine-learning algorithms for classification and traceability. In these applications, model generalizability across different datasets is a critical factor. During fermentation, brewing, or extraction, integrating spectral and ultrasonic signals into an online monitoring system allows real-time tracking of polyphenol content changes. The effective coordination of these multiple signals remains a key technical hurdle. For discriminating individual polyphenols and complex mixtures, functional probes and nanopores offer specific recognition capabilities that distinguish structurally similar compounds. Catechins are the major components of tea polyphenols, accounting for 60–80% of the total polyphenol content. Other plant phenolics contain similar catechol and pyrogallol groups and respond in a similar way in many colorimetric and electrochemical assays. Because of this structural similarity, it is difficult to tell tea-derived polyphenols apart from those of other plant sources. Future work should aim to develop recognition elements or signal patterns that can specifically identify tea polyphenols for authenticity testing and origin tracing.

## Figures and Tables

**Table 1:** Representative probe-based optical sensing technologies for TP detection.

Technique	Representative Systems	Detection Mechanism	Target Analyte	Advantages	Limitations	Ref.
Colorimetric sensing	Metal-ion complexation and nanozyme-catalyzed chromogenic reactions	Chromogenic reaction	Tannic acid; total TPs	Visual detection and simple operation	Limited sensitivity	[36,37]
Fluorescent sensing	Carbon quantum dots, Tb^3+^ complexes, and fluorescent recognition probes	Fluorescence quenching or recovery via TP–probe interactions	EGCG; gallate; total TPs	High sensitivity	Susceptible to photobleaching and environmental factors	[50,51,52,53]

**Table 2:** Representative electrochemical sensing technologies for TP detection.

Technique	Representative Systems	Detection Mechanism	Target Analyte	Advantages	Limitations	Ref.
Direct electrochemical oxidation	Bare or modified electrodes combined with CV or DPV	Anodic oxidation of phenolic hydroxyl groups at electrode surface	Total TPs; gallic acid; EGCG; catechins	Simple, reagent-free, rapid response	Poor reproducibility	[59,60,67,68]
Enzyme-mimetic electrochemical sensing	AuNPs/tyrosinase, cellophane/PPO, and GNRs/AgNPs/PPO composite electrodes	Enzyme-catalyzed oxidation of phenolic substrates	Gallic acid; catechol; total TPs	Enhanced catalytic activity, sensitivity, and selectivity	Enzyme instability, sensitive to pH and temperature	[29,62,61]
FIA coupled with electrochemical	GCE/GMC and DC-FBAS systems	Automated sample injection with electrochemical detection	Total TPs	High throughput	Complex instrumentation	[57,66]

**Table 3:** Spectroscopic and nondestructive methods for TPs detection.

Technique	Sensing Principle	Data Processing	Target Analyte	Advantages	Limitations	Ref.
Visible spectroscopy	Reflectance spectral information related to chemical bonds and functional groups	Ridge regression, RF	Total TPs	Rapid, non-destructive, low-cost portable devices	Limited information for quantification	[69]
Near-infrared spectroscopy	Overtones and combination vibrations of O–H, C–H, N–H bonds	PLSR, CARS-PLS, SVM, RF	Total TPs	Rapid, non-destructive, no reagents	Model Transferability issues	[70,71]
Hyperspectral imaging	Spectral reflectance combined with spatial information	SVR, RF, stacking ensemble	Total TPs	Integration of spectral and spatial information	Complex modeling	[78,79,80,84]
Ultrasonic sensing	Sound velocity changes with solute concentration	Temperature-compensated calibration	Total TPs	Unaffected by sample color and suitable for online monitoring	Non-specific signal; temperature-sensitive	[83]

## Data Availability

No new data were created or analyzed in this study. Data sharing is not applicable to this article.
